# Household catastrophic payments for tuberculosis care in Nigeria: incidence, determinants, and policy implications for universal health coverage

**DOI:** 10.1186/2049-9957-2-21

**Published:** 2013-09-17

**Authors:** Kingsley Nnanna Ukwaja, Isaac Alobu, Seye Abimbola, Philip Christy Hopewell

**Affiliations:** 1 Department of Internal Medicine, Federal Teaching Hospital, Abakaliki, Ebonyi State, Nigeria; 2 National Tuberculosis and Leprosy Control Programme, Ministry of Health, Abakaliki, Ebonyi State, Nigeria; 3 National Primary Health Care Development Agency, Abuja, Nigeria; 4 Francis J Curry International Tuberculosis Center, Division of Pulmonary and Critical Care Medicine, San Francisco General Hospital, University of California, San Francisco, CA, USA

**Keywords:** Cost analysis, Health policy, Tuberculosis, Regression analysis, Nigeria

## Abstract

**Background:**

Studies on costs incurred by patients for tuberculosis (TB) care are limited as these costs are reported as averages, and the economic impact of the costs is estimated based on average patient/household incomes. Average expenditures do not represent the poor because they spend less on treatment compared to other economic groups. Thus, the extent to which TB expenditures risk sending households into, or further into, poverty and its determinants, is unknown. We assessed the incidence and determinants of household catastrophic payments for TB care in rural Nigeria.

**Methods:**

Data used were obtained from a survey of 452 pulmonary TB patients sampled from three rural health facilities in Ebonyi State, Nigeria. Using household direct costs and income data, we analyzed the incidence of household catastrophic payments using, as thresholds, the traditional >10% of household income and the ≥40% of non-food income, as recommended by the World Health Organization. We used logistic regression analysis to identify the determinants of catastrophic payments.

**Results:**

Average direct household costs for TB were US$157 or 14% of average annual incomes. The incidence catastrophic payment was 44%; with 69% and 15% of the poorest and richest household income-quartiles experiencing catastrophic activity, respectively. Independent determinants of catastrophic payments were: age >40 years (adjusted odds ratio [aOR] 3.9; 95% confidence interval [CI], 2.0, 7.8), male gender (aOR 3.0; CI 1.8, 5.2), urban residence (aOR 3.8; CI 1.9, 7.7), formal education (aOR 4.7; CI 2.5, 8.9), care at a private facility (aOR 2.9; 1.5, 5.9), poor household (aOR 6.7; CI 3.7, 12), household where the patient is the primary earner (aOR 3.8; CI 2.2, 6.6]), and HIV co-infection (aOR 3.1; CI 1.7, 5.6).

**Conclusions:**

Current cost-lowering strategies are not enough to prevent households from incurring catastrophic out-of-pocket payments for TB care. Financial and social protection interventions are needed for identified at-risk groups, and community-level interventions may reduce inefficiencies in the care-seeking pathway. These observations should inform post-2015 TB strategies and influence policy-making on health services that are meant to be free of charge.

## Multilingual abstracts

Please see Additional file 1 for translations of the abstract into the six official working languages of the United Nations.

## Background

Globally, over 150 million people are pushed into poverty annually because they have to pay for health services directly [1]. To reduce these financial risks, the World Health Organization (WHO) has asked countries to put in place innovative health financing systems to ensure universal health coverage (UHC) [1]. As well as that, an increasing numbers of global health players have acknowledged the need to achieve UHC [1]. The attainment of UHC was one of the main recommendations following the 2012 Global Symposium on Health Systems Research [2], and in December 2012, the United Nations passed a draft resolution that would support UHC for people everywhere [3].

There is a strong association between poverty and ill-health [4]. In particular, poor and vulnerable groups are at an increased risk of tuberculosis (TB) disease and mortality [5,6]. The costs patients incur due to illness, including TB, remain central to the performance of health systems, particularly in terms of equity and coverage [1]. Excessive out-of-pocket (OOP) payments for health can discourage poor patients from seeking or continuing care [6,7]. In resource-poor countries, there is an urgent need to improve case-detection rates and treatment success of TB. Due to the strong link between poverty and TB, this will likely require reducing the economic burden of care.

Several studies have assessed patient and household costs of TB care [6-8]. These studies have two major limitations. First, costs of TB care were reported as averages and the economic impact of these payments were estimated using average incomes [6-8]. Although poor patients generally spend less on treatment compared to other economic groups, the amount spent constitutes a higher proportion of their income [9]. Thus, reports based on any average income figure will likely underestimate the economic impact of illness for patients with a relatively low economic status, which includes an important proportion of TB patients. Second, the extent to which TB expenditures risk sending a household into, or further into, poverty and its determinants, is unknown.

Although, the WHO recommends that national TB programs lower patient costs of TB care-seeking through removal of user fees, decentralization of services, community-based care, and free diagnosis and treatment [5,10], an important question that still needs to be answered empirically is whether household TB expenditures are large enough to require consumption of other goods to be sacrificed [11]. The basis of this concept is that such high level of spending must be at the expense of the consumption of other goods and services in the short- or long-term. This level of payments is often qualified as catastrophic [12-14]. Catastrophic healthcare expenditures have been defined by a rather arbitrary fraction of the total household income (>10%) or of the total income net of subsistence expenditure (≥40%) during a reference period [11-15]. The second definition better captures the ability to pay and better distinguishes between the rich and the poor [15,16].

No studies have fully applied the same concept to assess the extent of catastrophic payments for TB care. Using data from a recent project on the economic impact of TB [17], and applying the same underlying conceptual framework as used for catastrophic health expenditures, this study investigated the incidence, intensity, distribution and correlates of catastrophic payments for TB care, and policy implications for TB care and other primary care services.

## Methods

### Data

The data used was obtained from a recent survey of 452 pulmonary TB patients sampled from three rural health facilities in Ebonyi State, Nigeria [17]. The survey’s design is described below.

### Study setting

Ebonyi State, located in the southeastern region of Nigeria has 73.6% of its 2.5 million population living below the poverty line [18]; and 75% of its inhabitants live in rural areas [19]. Because of the low availability of functional public sector health services, and the inability of the health system to recruit and retain health workers in rural settings of Ebonyi State, several not-for-profit mission (private) hospitals provide basic and secondary health services [17]. Since the Nigeria National TB Control Programme (NTP) was started, these not-for-profit mission hospitals and public (tertiary, secondary, and primary) health facilities have been involved in the decentralization of TB control services. Ebonyi State is divided into three geopolitical zones. The study was conducted in three rural hospitals (one public and two not-for-profit mission hospitals), one from each zone. Each of the study hospitals is a zonal referral center for TB services, and together, they accounted for 70% of TB case notification in Ebonyi State in 2009 [20].

Tuberculosis treatment at the hospitals surveyed was based on the community-based directly observed treatment short-course (community-DOTS) strategy in accordance with the guidelines of the NTP. Briefly, newly-diagnosed TB patients are treated with daily rifampicin, isoniazid, pyrazinamide, and ethambutol for two months of intensive treatment; followed by ethambutol and isoniazid for six months. During the first month of intensive phase of treatment for hospitalized patients, anti-TB medications are given and directly supervised at each study hospital by a medical staff of the hospital. Non-hospitalized TB patients attend weekly drug collection visits for their anti-TB medications. Therapy for patients treated on an outpatient basis was observed by a family member (DOTS-supporter) and/or a volunteer community health worker (CHW). At each drug collection visit, TB patients are required to come with their DOTS-supporter and the empty packs/sachets of used drugs. The activities of the DOTS-supporter and the CHW are monitored by the district TB control supervisors.

In Nigeria, households provide 70% of their total health expenditures and 90% of these occur through OOP payments [21]. Although a national policy that exempts TB patients/suspects from paying for consultations, laboratory tests, TB drugs, and hospitalization does exist, it was not effective at the not-for-profit mission hospitals surveyed. All patients who visited the mission hospital irrespective of their illness are required to pay a nominal consultation fee. Tuberculosis patients requiring hospital admissions were told to pay additional fees for accommodation and feeding, but TB laboratory tests and treatment are provided free of charge. At the public sector hospital surveyed, TB services (consultations, diagnostic tests, drugs, and hospitalization) are provided free of charge. However, feeding was not provided for TB patients who were hospitalized. There is no risk-pooling mechanism in financing health care as the National Health Insurance Scheme (NHIS) in Nigeria currently covers only federal government workers, who are <5% of the population [22].

### Study design

A cross-sectional study of new pulmonary TB patients was conducted between January and August 2011 to assess the costs of TB to patients and their households from the onset of the TB illness to diagnosis to the end of the intensive phase of treatment.

### Participants

Only adult TB patients diagnosed based on sputum smear results with or without chest X-ray, and who had completed at least one month but less than three months of treatment i.e., between the 5th and 11th weekly drug collection visits, were included. Extra-pulmonary TB patients, previously treated TB patients, and those with other co-morbidities except human immunodeficiency virus (HIV) were excluded. All the patients received community-DOTS.

### Sample size and sampling strategy

Assuming that 50% of households with TB patients incur catastrophic OOP payments for TB care at 95% confidence level and an absolute sampling error of 5%, a minimum of 378 patients were needed. The sample size was increased to 480 to account for potential refusals and to allow additional variables in the logistic regression analysis. Consecutive eligible patients were interviewed at each study facility until the required sample size was reached.

### Data collection

A standardized questionnaire (tool to estimate patient cost) from the WHO/Stop-TB partnership was used for the survey [23]. The questions included a detailed list of the expenses that might be incurred by TB patients in their pathway to care i.e., from illness onset to visit to any health provider(s) to diagnosis to treatment. Also, there were other questions which also quantified the incomes and food expenditures of the patients and their households before the onset TB symptoms, and at the point when TB was diagnosed and treatment was started [23]. The annual income of each patient’s household was estimated from their mean monthly income reported at the time of their diagnosis.

Data was collected by two CHWs with Bachelor’s degrees. All the patients were interviewed in their preferred language during their drug collection visit to the study hospitals and each interview lasted an average of 30 minutes. Quality control was carried out by the respective district TB control officers charged with guiding and inspecting every step of the survey, and was also carried out weekly by the principal investigator.

### Measuring catastrophic payments

Using household direct cost for TB care and income data, we analyzed the incidence of household catastrophic payments and its determinants. Patient direct costs include the medical OOP payments (e.g. consultation fees, laboratory tests, drugs from any source, and hospital care) and the non-medical OOP expenses (e.g. the costs of travel, services provided by traditional healers, any food supplements, and coping costs). For each household, the total household direct payment for TB care was the sum of the direct patient costs and the direct DOTS-supporter costs (see Table 1).

**Table 1:** Cost definitions used in the study

**Variable**	**Definition**
***Direct cost***	Out-of-pocket payment for TB services and those incurred in the pathway to care to access the service. These include user fees, consultation fees, laboratory test fees, travel costs, food costs, and other costs.
***Household cost***	For each patient, this is the sum of patient direct pre-diagnosis cost, direct coping cost, direct post-diagnosis cost, and direct DOTS-supporter cost.
***Coping cost***	Costs incurred by patients who attempted to cope with the costs of TB care by borrowing money or selling their assets. It is estimated as a sum of interest paid for loans and/or the difference in the market value of assets sold.
***DOTS-supporter cost***	The direct costs incurred by family members looking after/accompanying the patient during care.
***Pre-diagnosis/ diagnosis cost***	The direct costs incurred in the period between the onset of symptoms up until the diagnosis of TB.
***Post-diagnosis cost***	The direct costs incurred in the period from the start to the completion of the intensive phase of treatment (approximately two months).

TB = tuberculosis; DOTS = directly observed treatment short-course strategy.

The incidence of catastrophic payments is estimated from the fraction of a population sample with healthcare costs as a share of the total income or non-food expenditure exceeding the chosen threshold. Most previous studies reported that an OOP payment greater than 10% of the total household income is catastrophic [12], while the WHO and World Bank defined total OOP payments equaling or exceeding 40% of the non-food household income (capacity to pay) as catastrophic [13-15]. The WHO approach is the widely accepted method for measuring the incidence of catastrophic payments [13-16]. In this study, catastrophic payments for TB care was estimated using the two measurement methods, and sensitivities of varying thresholds were determined. These two concepts are illustrated in the conceptual framework detailed in (see Table 2).

**Table 2:** Conceptual framework

**Out-of-pocket (direct) costs for tuberculosis care**	**Economic burden**	**Measurement tools**	**Indicators**	**Interpretation and implications**
	Catastrophic payments (I)	Percentage of direct costs exceeding 10% of the household income	Incidence (%) and intensity using various income thresholds [5%, 10%, 15%, 20%, 25%]	Which rates of catastrophic costs using income threshold agrees with the burden estimated using ≥40% of non-food expenditure (II)
	Catastrophic payments (II)	Percentage of direct costs greater than or equal to 40% of the non-food income	Incidence (%) and intensity using various thresholds of non-food expenditure [≥15%, ≥25%, ≥40%]	Distribution of catastrophic costs of care across patient characteristics and its determinants

Households that earned less than the median annual income reported during the study ($1040) were classified as poor households and those that earned more than the median were classified as less-poor. All costs were captured in Nigeria Naira (N) and converted to the 2011 US$ rate (1 US$ = N150).

### Data analysis

The data were double entered, checked, and analyzed using Epi Info 3.4.1 (CDC, Atlanta, GA USA). The normality of direct cost data distribution was assessed using visual inspection of graphs created using the data and Chebyshev’s rule of frequency distribution. Direct costs were normally distributed. Continuous variables were summarized as means (± standard deviation [SD]) while categorical variables were summarized as proportions (%). Categorical group comparisons were made using the Chi-squared test. Odds ratios and their 95% confidence intervals (CIs) were estimated using multivariable logistic regression analysis, with household catastrophic payments (i.e., ≥ 40% of capacity to pay) as an outcome variable. The likelihood ratio test was used to assess the association between explanatory variables and household catastrophic payments. We performed a stratified analysis to determine the occurrence of interaction and confounding between the outcome variable and explanatory variables. Multivariable models were then constructed, including all variables of clinical importance and all with bivariate P < 0.25. A backward elimination approach was used to find the best model. All P-values were bidirectional and significance was set at a P-value of less than 0.05.

### Ethical issues

Written informed consent was sought from all study participants. The research protocol was approved by the Ebonyi State University Teaching Hospital Health Research and Ethics Committee.

## Results

### Household income and OOP payments for TB

Complete data from 452 patients and their households were available for our analysis. The average household annual income was US$1123 of which an average of 47% was spent on food. Mean direct household payments for TB were US$157 or 14% of the average annual income. About 45% of this amount was spent before TB was diagnosed and 25% each during treatment and through coping strategies adopted by the household (see Table 3). Drugs from several sources (35%), laboratory tests (30%), and transportation (21%) during the care-seeking pathway accounted for most payments from the onset of illness up until diagnosis. Transportation (70%), hospitalization (15%), and feeding (13%) accounted for the majority of costs during treatment.

**Table 3:** Household direct payments for TB care in Nigeria, 2011

**Source of direct cost**	**Mean costs (± SD) in US$**	**Percentage of mean annual household income (%)**
***Patient pre-diagnosis***	70 (±48.4)	6.2
***Patient post-diagnosis***	40 (±32.2)	3.6
***DOTS-supporter***	7 (± 6.5)	0.6
***Coping cost***	40 (±38.5)	3.6
***Total***	157	14

US$ = United States dollar.

Mean annual household income at TB diagnosis = US$1123.

SD = Standard deviation.

*Note:* Based on a currency exchange rate of 150 Nigeria Naira to US$1.00.

### Catastrophic payment in different income groups

The rate of catastrophic payments for TB was inversely associated with the household income level. Ninety-five percent of households spent at least 5% of their income on TB treatment (direct cost), 65% spent at least 10%, and 37% spent at least 15%. Defining catastrophic payments using the current WHO definition, 44% of households incurred catastrophic payments for TB care. The average OOP payments and capacity to pay rose steadily with rising income quartile i.e., the wealthier the quartiles, the higher the OOP payments and capacity to pay. However, the economic burden faced by households in the richer income quartiles was proportionally lower due to their higher capacity to pay (see Table 4). Thus, although the average direct costs of TB care increased from US$128 among the households in the lowest income quartile to US$208 among those in the highest income quartile (Chi-square for trend 39, *P* < 0.001), the proportion of households who incurred catastrophic payments decreased from 69% among households belonging to the poorest income quartile to 15% among those belonging to the richest income quartile (Chi-square for trend 148, *P* < 0.001).

**Table 4:** Distribution of household direct costs and incidence of catastrophic payments for TB across income quartiles, Nigeria, 2011

**Indicator**	**Income quartile**^**a**^				
	**Q1**	**Q2**	**Q3**	**Q4**	**All**
***Frequency***	174	66	134	78	452
***Average direct costs of TB care (US$)***^***b***^	128	144	158	208	157
***Average annual household income (US$)***	644	880	1313	2071	1123
***Direct costs share of household income (%)***	20	16	12	10	14
***Average capacity to pay***^***c ***^***(US$)***	319	320	675	985	540
***Households with catastrophic expenditure (%)***					
*I = >10% of household income*	88	100	45	31	65
*II = ≥40% of capacity to pay*	69	46	27	15	44

US$ = United States dollar.

^a^Quartile 1 is the poorest and Quartile 4 is the wealthiest.

^b^Based on a currency exchange rate of 150 Nigeria Naira to US$1.00.

^c^Capacity to pay = household income minus food expenditure (income after expenditures for food).

### Threshold level for catastrophic payment

The sensitivity of the analysis to different threshold levels of catastrophic payments was tested as shown (see Table 5). The head count is the percentage of households whose direct costs, expressed as a proportion of income, exceeds a given discretionary fraction of their income, z. The mean gap is the average amount by which payments, as a proportion of income, exceed the threshold z. The head count (a measure of incidence) and mean gap are related through the mean positive gap (a measure of intensity), which is defined as the head count being a fraction of the mean gap. The table shows that as much as 44% of the sample recorded OOP payments for TB as a proportion of income which exceeded 40% of their capacity to pay. The related mean gap was 8.3%, which means that on average, TB-related expenditures were 8.3% higher than the 40% threshold.

**Table 5:** Incidence and intensity of catastrophic payments for TB care, Nigeria 2011

**Catastrophic payment**	**Threshold level ( *z *)**				
	**5%**	**10%**	**15%**	**25%**	**40%**
***Out-of-pocket TB spending: as a share of total expenditure***					
*Head count (%)*	95	65	37	9	
*Mean gap (%)*	7.5	6.0	4.6	2.1	
*Mean positive gap (%)*	7.9	9.3	12.5	22.9	
***as a share of non-food expenditure***					
*Head count (%)*			84	68	44
*Mean gap (%)*			12.3	10.7	8.3
*Mean positive gap (%)*			14.8	15.9	19.1

### Catastrophic payment distribution

We compared catastrophic TB-related expenditure rates among various patients and households with different characteristics (see Table 6). Households with a male patient had a greater risk of experiencing catastrophic payments than those with a female patient. Urban households had a higher risk of experiencing catastrophic payments than rural households. Poor households had a greater risk of incurring catastrophic payments than less-poor households. Patients who received care at a private facility had a higher risk of catastrophic payments than those who were treated at a public facility. Also, households with a patient who was the household primary income earner had a higher risk of incurring catastrophic payments than those who were not.

**Table 6:** Relationship between patients’ characteristics, average household direct costs, and rate of catastrophic payments for TB, Nigeria, 2011

**Variable**	**Direct costs (US$)**	**T-test**	***P***	**Rate of catastrophic payment n (%)**	**X**^**2**^	***P***
**Age (years)**		6.01	<0.001		0.024	0.9
≤ 40	144			150 (43.6)		
> 40	197			48 (44.4)		
**Gender**		4.2	<0.001		26.1	<0.001
Male	171			138 (54.3)		
Female	138			60 (30.3)		
**Residence**		3.1	0.002		69	<0.0
Urban	180			78 (81.3)		
Rural	150			120 (33.7)		
**Education**		0.88	0.4		40.3	<0.001
No formal education	153			42 (24.7)		
Formal education	159			156 (55.3)		
**Primary income earner**		4.1	<0.001		56.3	<0.001
Yes	174			126 (63.6)		
No	143			72 (28.3)		
**Household income level**		2.6	0.01		72.8	<0.001
Poor	144			120 (69)		
Less-poor	165			78 (28.1)		
**Category of facility**		0.7	0.47		37.6	<0.001
Public	161			24 (20)		
Private	155			174 (52.4)		
**Smear status**		0.02	0.98		0.66	0.42
Smear positive	157			162 (44.8)		
Smear negative	157			36 (40)		
**HIV status**		4.4	<0.001		1.3	0.25
Positive	168			66 (45)		
Negative	131			132 (42)		

HIV = human immunodeficiency virus.

### Determinants of catastrophic payment

Logistic regression analysis yielded a wide range of independent determinants of catastrophic TB-related expenditure (see Table 7). Households with a male patient, a patient >40 years, a patient with formal education (i.e., completed at least six years of schooling), or who was the primary income earner for their household were more likely to experience catastrophic payments. Urban residence, care at a private facility, and HIV co-infection were other independent determinants of catastrophic payments for TB care.

**Table 7:** **Determinants of catastrophic payments**^**a **^**for TB care, Nigeria, 2011**

**Variable**	**Coeff**	**Standard error**	**P-value**	**Odds ratio (95% CI)**
***Older age (>40 years)***	1.368	0.35	<0.001	3.9 (2.0 – 7.8)
***Male gender***	1.11	0.28	<0.001	3.0 (1.8 – 5.2)
***Urban residence***	1.34	0.36	<0.001	3.8 (1.9 – 7.7)
***Formal education***	1.55	0.33	<0.001	4.7 (2.5 – 8.9)
***Care at private facility***	1.10	0.36	0.003	2.9 (1.5 – 5.9)
***Poor patients***	1.91	0.31	<0.001	6.7 (3.7 – 12.4)
***Household primary earner (patient)***	1.33	0.28	<0.001	3.8 (2.2 – 6.6)
***HIV (positive) status***	1.13	0.30	<0.001	3.1 (1.7 – 5.6)
***Smear positive TB***	0.10	0.35	0.77	1.1 (0.6 – 2.2)
***Constant***	2.11	0.52	<0.001	

Coeff. = coefficient expressed in logits; CI = 95% confidence interval for the odds ratio, TB = tuberculosis, HIV = human immunodeficiency virus.

## Discussion

The incidence and intensity of catastrophic payments for TB care could provide an insight into the level of financial protection a healthcare financing system affords its population [24]. It shows the financial burden faced by households of TB patients, and the economic and information barriers that impair prompt and appropriate TB-care seeking. Thus, although TB care is supposedly free of charge, patients/households have to make significant OOP payments in their pathway to diagnosis and treatment. In this study, incidence of catastrophic payment was 44%. The rates observed are higher than those estimated for catastrophic health expenditure, in general, in African and other low-income countries [24,25].

About half of the costs incurred by the patients were made before diagnosis. This suggests that TB patients incur substantial OOP payments before reaching a health facility where the disease can be diagnosed. This high pre-diagnosis cost may be due to poor community knowledge of TB resulting in long delays and inappropriate care seeking at informal providers for TB symptoms [26]. Furthermore, it may be that the informal/non-NTP providers kept the patients for profits, or had low capacity to diagnose and refer TB suspects to a DOTS facility. Reducing the high pre-diagnosis costs may require improving community knowledge of TB, further decentralization of TB services to rural communities, and involving informal/non-NTP providers in TB service delivery.

### Socioeconomic status and catastrophic payment

Our findings showed that economic status was a key determinant of catastrophic payments. This result is similar to what was previously documented [24,25]. Payments for TB care reduced more of the average capacity to pay/income of the two poorest income groups (Q1 and Q2). As previously reported [13], three conditions are required for catastrophic payments to occur: the availability of health services requiring payment, low capacity to pay, and the absence of prepayment mechanisms. Health services for TB are available in the setting, but there is high usage of the private/informal sector requiring payments [26]. The use of informal providers have been shown in other studies in southeast Nigeria and elsewhere to be common among the poor, and may contribute to their high rates of catastrophic health payments [27,28]. Although TB care at the public sector is free, inequities in the distribution of effective public sector services between the rural and urban areas as well as “under-the-table payments” may account for high rates of catastrophic payments among those who received care in the rural public sector. With about 70% of Nigerians living on less than US$1 per day [18], high poverty rates may account for the low capacity to pay among households with a TB patient. In addition, their more costly and tortuous care-seeking pathway [26,29], results in substantial OOP payments as most households are not covered by prepayment mechanisms [22].

### Factors associated with catastrophic payments

Logistic regression analysis showed that demographic factors such as age, gender, education, being the primary household earner, and residence independently influenced the risk of catastrophic payment for TB care. Households with a male patient, a patient over 40 years, a patient with formal education, or a patient who is the primary earner were more likely to incur catastrophic payments. Patients from urban households incurred higher rates of catastrophic payments because the study health facilities were located in the rural area thus transportation costs to the rural hospitals may be the main culprit. Households with male patients are more affected because in the setting, men are the primary income earners. The high rate of catastrophic payments in households with older patients may occur because with increasing age, patients are more likely to visit a hospital with a DOT-supporter accounting for higher costs.

HIV co-infection was also found to be an important determinant of catastrophic payments. A previous study suggests that TB/HIV co-infection could be less costly to patients because of TB/HIV collaborative activities [30]. However, we found that households with a HIV-infected patient were three times more likely to incur catastrophic payments than those without. Thus, households with a HIV-infected patient in similar settings should be targets for financial protection mechanisms.

### Limitations of the analysis

This study had some limitations. First, it captures the incidence of catastrophic OOP payments of households with TB patients who sought care and did not consider those who need services but could not afford them. This could lead to underestimation of the incidence and intensity of catastrophic payments. Second, households may suffer additional financial problems because of foregone income associated with TB disease [6]. We deliberately focused only on estimating the financial catastrophe associated with OOP payments made for TB care because high OOP payments may discourage early care seeking or push the patient/household into poverty [1]. Other potential limitations include the use of cross-sectional survey data and recall bias. Data were collected when the patients had recently started treatment and should have a better recall of their care-seeking pathway. Further qualitative research will complement and enrich the findings of this study.

## Conclusions

In conclusion, 44% of households incurred catastrophic payments for TB care, and the proportion of households who incurred catastrophic payments for TB care was inversely associated with their household income. Our study points to four important implications for health policymakers. First, Nigeria and other high-burden TB countries must move away from OOP payments for health care. There is a need to institute financial protection against catastrophic payments through prepayment mechanisms. This may be achieved through a mix of tax-based, social health, and community-based insurance schemes. Second, policymakers can use the findings in this paper to better target beneficiaries of financial risk-protection strategies. Importantly, there is a need to develop strategies to better protect households with TB patients who are older than 40 years, male, educated, primary household earners, and HIV infected against catastrophic payments associated with paying for TB care. Third, there is a need to reduce the cost of TB care by curbing unnecessary pre-diagnosis costs (e.g., from inappropriate care-seeking and irrational drug use), and strengthening lower‒level providers. This can be achieved through extensive community engagement mechanisms to restructure the institutional arrangements around the health service uptake through education, and empowerment of informal providers and their use as referral points in the service chain.

Finally, the current low TB detection rate in high-burden countries implies that current approaches to reduce patient costs of care are not enough to prevent TB-affected households from incurring catastrophic payments. It also suggests that very poor patients may not be able to afford care. Decision makers may need to institute social protection interventions such as food or cash transfers, and microcredit schemes for TB patients in high-burden countries. Our observations should not only inform the ongoing consultations on sustainable post-2015 strategies for TB care and control, but also influence the thinking of national and global health policymakers on how to plan for free health services to ensure that these services are actually free of charge.

## Competing interests

The authors declare that they have no competing interests.

## Authors’ contributions

KNU led the study from conception, design and data collection to the drafting of the article. IA contributed to the design, interpretation of data, and drafting of the article. SA contributed to data analysis, and interpretation and drafting of the article. PCH contributed to the conception, design, and revising the manuscript critically for important intellectual content. All authors gave final approval.

## Authors’ information

KNU is an Internal Medicine resident clinician and a facilitator for the National TB and Leprosy Control Programme in Ebonyi State. IA is a public health physician and the program officer of the National TB and Leprosy Control Programme in Ebonyi State. SA is an epidemiologist and a project fellow of the National Primary Health Care Development Agency, Abuja, Nigeria. PCH is a Professor in residence, Division of Pulmonary and Critical Care Medicine, San Francisco General Hospital, and the Principal Investigator of the Francis J Curry International Tuberculosis Center,

## Supplementary Material

Additional file 1 Multilingual abstracts in the six official working languages of the United Nations. Click here for file
