# Supplementary material for: Household catastrophic payments for tuberculosis care in Nigeria: incidence, determinants, and policy implications for universal health coverage
Source: Infect Dis Poverty. 2013 Sep 17;2:21. doi: 10.1186/2049-9957-2-21 (PMC3848689; doi:10.1186/2049-9957-2-21)
Supplement: Additional file 1 — Multilingual abstracts in the six official working languages of the United Nations. [file 2049-9957-2-21-S1.pdf]

Translation of the abstract into the six official working languages of the United Nations

المدفوعات المنزلية الكارثية لرعاية مرضى السل في نيجيريا: الإصابة، المحددات، والآثار المترتبة على السياسات للتغطية الصحية الشاملة

Kingsley Nnanna Ukwaja, Isaac Alobu, Seye Abimbola, Philip Christy Hopewell

ملخص

خلفية : إن الدراسات حول التكاليف التي يتكبدها مرضى السل (TB) في الرعاية هي محدودة حيث يتم الإبلاغ عن هذه التكاليف كقيم وسطية، ويقدر الأثر الاقتصادي للتكاليف على أساس متوسط دخل المريض / الأسرة. متوسط النفقات هذه لا تمثل الفقراء لأنهم ينفقون بشكل أقل على العلاج مقارنة بالمجموعات الاقتصادية الأخرى . وبالتالي، فمن غير المعروف ما هو مدى الخطر الذي تشكله نفقات السل في وضع الأسر في الفقر أو المزيد منه و محدداته. ولقد قمنا بتقييم حالات ومحددات المدفوعات المنزلية الباهظة لرعاية مرضى السل في المناطق الريفية في نيجيريا.

الأساليب : تم الحصول على البيانات المستخدمة من دراسة استقصائية من 452 من مرضى السل الرئوي تم اختيارهم من ثلاثة مرافق صحية ريفية في ولاية إيبوني، نيجيريا. باستخدام التكاليف المنزلية المباشرة وبيانات الدخل، قمنا بتحليل حالات المدفوعات الباهظة المنزلية باستخدام النسبة التقليدية وهي > 10٪ من دخل الأسرة و ≥ 40٪ من الدخل غير الغذائي كمستويات دنيا، على النحو الموصى به من قبل منظمة الصحة العالمية. استخدمنا تحليل الانحدار اللوجستي لتحديد محددات المدفوعات الباهظة.

النتائج: كان متوسط التكاليف المنزلية المباشرة لمرض السل 157 دولار أمريكي أو 14٪ من متوسط الدخل السنوي. وكان سداد الحالات الباهظة 44٪، مع 69٪ و 15٪ من أفقر وأغنى الأسر ذات دخل الشرائح الربعية التي تعاني من أنشطة كارثية، على التوالي. وكانت المحددات المستقلة للمدفوعات الباهظة: العمر> 40 سنة (نسبة الأرجحية المصححة [aOR] 3.9 ؛ فاصل الثقة 95٪ [CI]، 2.0، 7.8)، جنس الذكور (منطقة المسؤولية 3.0؛ CI 1.8، 5.2)، الإقامة في الحضر (AOR 3.8 ؛ CI 1.9، 7.7)، والتعليم الرسمي (منطقة المسؤولية 4.7؛ CI 2.5، 8.9)، والرعاية في منشأة خاصة (منطقة المسؤولية 2.9؛ 1.5، 5.9)، الأسر الفقيرة (6.7 منطقة المسؤولية؛ CI 3.7، 12)، الأسر التي يكون فيها المريض هو كاسب الدخل الأساسي (AOR 3.8؛ CI 2.2، 6.6])، والإصابة المصاحبة للفيروس المسبب للإيدز (AOR 3.1؛ CI 1.7، 5.6).

والخلاصة: لا تكفي استراتيجيات خفض التكاليف الحالية لمنع الأسر من تكبد تكاليف شخصية باهظة لرعاية مرضى السل. وهناك حاجة إلى تدخلات للحماية المالية والاجتماعية للفئات المعرضة للخطر التي تم تحديدها، وإلى تدخلات على مستوى المجتمع المحلي للحد من أوجه القصور في مسار التماس الرعاية. وينبغي إيصال هذه الملاحظات إلى استراتيجيات السل ما بعد عام 2015 والتأثير في صنع سياسات الخدمات الصحية التي يراد لها أن تكون مجانية.

Translated from English version into Arabic by Ibtihal83, through

TRANSLATORS WITHOUT BORDERS

尼日利亚结核病治疗的家庭灾难性支出：发生率、影响因素和全民健康覆盖的政策影响

Kingsley Nnanna Ukwaja, Isaac Alobu, Seye Abimbola, Philip Christy Hopewell

摘要

引言：结核病护理患者支出研究的局限性表现在以下两个方面。一是报道的费用为治疗费用的平均值，根据患者/家庭的平均收入评估治疗费用的经济影响。二是平均支出不能代表贫困群体，因为与其他经济群体相比，他们花费治疗费用较少。因此，尚不清楚结核病支出导致家庭贫困或加剧贫困的风险程度及其影响因素。本研究在尼日利亚农村地区对结核病治疗家庭灾难性支出的发生率和影响因素进行评估。

方法：数据来自尼日利亚埃邦伊州的 3 个农村医疗机构的 452 例肺结核病患者。使用家庭直接成本和收入数据分析家庭灾难性支出的发生率，根据 WHO 推荐的标准，以传统的>10%家庭收入和≥40%非食品收入为阈值。利用 logistic 回归分析确定灾难性支付的影响因素。

结果：结核病家庭平均直接成本为 157 美元或年均收入的 14%。家庭灾难性支出发生率为 44%，最贫穷和最富有家庭灾难性支出发生率分别为 69%和 15%。灾难性支付的独立影响因素有：年龄>40 岁 (aOR 3.9，CI 2.0，7.8)、男性(aOR 3.0; CI 1.8, 5.2)、城镇户籍(aOR 3.8; CI 1.9, 7.7)、正规教育(aOR 4.7; CI 2.5, 8.9)、在私人机构接受治疗(aOR 2.9; 1.5, 5.9)、贫困家庭(aOR 6.7; CI 3.7, 12)、患者为主要劳动力的家庭(aOR 3.8; CI 2.2, 6.6])和 HIV 联合感染(aOR 3.1; CI 1.7, 5.6)。

结论：目前低成本策略对于防止结核病治疗产生灾难性支出是不够的。需要财政和社会保障确定高危人群，社区一级的干预可能会提高就诊途径的效率。这些结果供 2015 年后结核病策略和卫生服务政策的制定免费使用。

Translated from English version into Chinese by Yang Pin, through

CHINESE CENTER FOR DISEASE CONTROL AND PREVENTION
NATIONAL INSTITUTE FOR PARASITIC DISEASES

Dépenses catastrophiques des ménages en soins contre la tuberculose au Nigéria: incidence, déterminants et implications politiques pour la couverture de santé universelle

Kingsley Nnanna Ukwaja, Isaac Alobu, Seye Abimbola, Philip Christy Hopewell

Résumé

Contexte : Les études consacrées aux frais supportés par les patients traités contre la tuberculose sont limitées puisque ces coûts sont présentés sous forme de moyennes et que l'impact économique de ces coûts est estimé sur base des revenus moyens des patients/ménages. La moyenne des dépenses ne représente pas les pauvres car ils dépensent moins pour les traitements comparés à d'autres groupes économiques. Ainsi, la mesure dans laquelle les dépenses liées à la tuberculose risquent de basculer les ménages dans/plus loin dans la pauvreté et ses causes déterminantes est inconnue. Nous avons évalué l'incidence et les facteurs déterminants des dépenses catastrophiques des ménages en soins contre la tuberculose dans le Nigéria rural.

Méthodes : Les données utilisées ont été obtenues sur base d'un sondage effectué auprès de 452 patients présentant une tuberculose pulmonaire et sélectionnés dans trois installations sanitaires rurales de l'État d'Ébonyi au Nigéria. En utilisant les données relatives aux coûts directs et aux revenus des ménages, nous avons analysé l'incidence des dépenses catastrophiques des ménages en utilisant comme seuils, le traditionnel >10% du revenu du ménage et le ≥40% du revenu non-alimentaire, comme le recommande l'OMS. Nous avons utilisé l'analyse logistique de régression pour identifier les déterminants des dépenses catastrophiques.

Résultats : Les coûts directs moyens des ménages pour les soins contre la tuberculose s'élevaient à 157 $ US ou 14% des revenus annuels moyens. L'incidence de paiement catastrophique était de 44 % ; avec 69 % et 15 % des quartiles de revenu des ménages les plus pauvres et les plus riches connaissant respectivement une activité catastrophique. Les déterminants indépendants des paiements catastrophiques étaient : âge >40 ans (quotients de probabilité ajustés [aOR] 3.9; 95% intervalle de confiance [CI], 2.0, 7.8), sexe masculin (aOR 3.0; CI 1.8, 5.2), lieu de résidence (aOR 3.8; CI 1.9, 7.7), enseignement formel (aOR 4.7; CI 2.5, 8.9), soins dans un établissement privé (aOR 2.9; 1.5, 5.9), ménage pauvre (aOR

6.7; CI 3.7, 12), ménage dans lequel le patient est le principal soutien économique (aOR 3.8; CI 2.2, 6.6]), et la co-infection par le VIH (aOR 3.1; CI 1.7, 5.6).

Conclusion : Les stratégies actuelles de réduction des coûts ne sont pas suffisantes pour empêcher les ménages d'être soumis à des dépenses catastrophiques en soins contre la tuberculose. Les interventions financières et sociales de protection sont nécessaires pour les groupes à risques identifiés et les interventions au niveau de la communauté peuvent réduire les inefficacités dans l'accès aux soins. Ces observations devraient être utilisées pour les stratégies contre la tuberculose postérieures à 2015 et influencer l'élaboration des politiques en matière de services de santé qui devraient être gratuits.

Translated from English version into French by Laetitia Michel, through

TRANSLATORS
WITHOUT BORDERS

Катастрофический уровень выплат на лечение туберкулеза в Нигерии: частота явления, показатели определения и сущность политики, направленной на всеобщее медицинское страхование

Кингли Нанна Аквая (Kingsley Nnanna Ukwaja), Исаак Алобу (Isaac Alobu), Сейе Эбимбола (Seye Abimbola), Филип Кристи Хоупвелл (Philip Christy Hopewell)

Реферат

Обоснование. Исследования стоимости лечения туберкулеза (ТБ) для пациентов содержат сведения о средних цифрах расходов на лечение, а экономическое влияние таких расходов рассчитано на основании данных о средних доходах пациента или семьи. Показатели средней стоимости лечения не отражают затрат бедного пациента или бедной семьи, потому что по сравнению с другими экономическими группами населения они тратят меньше денег на лечение. В связи с этим неизвестно, насколько велика степень риска относительно влияния стоимости лечения ТБ на вероятность перейти в группу населения с доходами ниже уровня бедности или дальнейшего снижения уровня жизни, и какие существуют методы определения таких рисков. Мы провели оценку частоты и показателей катастрофического уровня выплат на лечение ТБ для семьи в сельской местности Нигерии.

Методы. Приведенные цифры были получены из анкет, заполненных 452 пациентами с легочной формой ТБ в трех сельских медицинских учреждениях штата Эбоньи в Нигерии. Пользуясь данными о прямых затратах семьи на лечение и доходе семьи, мы провели анализ частоты катастрофического уровня выплат для семьи, применяя в качестве порогового значения традиционные величины (согласно рекомендациям Всемирной организации здравоохранения): >10% дохода семьи и ≥40% дохода, не предназначенного для продуктов питания. Мы применяли модель логистической регрессии для определения показателей катастрофического уровня выплат.

Результаты. Средний показатель прямых затрат семьи на лечение ТБ составил 157 долларов США или 14% среднего показателя ежегодного дохода. Частота катастрофического уровня выплат равнялась 44%; при этом квартили доходов у 69% наиболее бедных семейств и 15% наиболее богатых семейств претерпели катастрофические изменения. Независимыми показателями для определения

катастрофического уровня выплат являлись: возраст >40 лет (скорректированное отношение шансов [СОШ] 3,9; 95% доверительный интервал [ДИ], 2,0, 7,8), мужской пол (СОШ 3,0; ДИ 1,8, 5,2), проживание в сельской местности (СОШ 3,8; ДИ 1,9, 7,7), уровень официального образования (СОШ 4,7; ДИ 2,5, 8,9), лечение в частном медицинском учреждении (СОШ 2,9; ДИ 1,5, 5,9), бедственное положение семьи (СОШ 6,7; ДИ 3,7, 12), семья, в которой пациент является основным источником дохода (СОШ 3,8; ДИ 2,2, 6,6]) и сопутствующая ВИЧ инфекция (СОШ 3,1; ДИ 1,7, 5,6).

Вывод. Стратегические методы, используемые в настоящее время для снижения расходов на лечение ТБ, являются недостаточно эффективными, чтобы не допустить катастрофического уровня оплаты стоимости лечения, тяжесть которой всецело лежит на самих семьях («из своего кармана»). Для указанных групп риска необходимо вмешательство финансовых управлений и служб социальной защиты, и вмешательство на уровне сообщества может повысить обращаемость пациентов и эффективность мероприятий при оказании медицинской помощи. Эти наблюдения должны предоставить информацию для стратегий, направленных на борьбу с ТБ и разрабатываемых на период с 2015 г., а также повлиять на лиц, создающих политику в области медицинских услуг, которые должны быть бесплатными.

Translated from English version into Russian by Irina Zayonchkovskaya, through

TRANSLATORS
WITHOUT BORDERS

Pagos catastróficos de las familias para atención de la tuberculosis en Nigeria: incidencia, determinantes e implicaciones de las políticas para la cobertura universal de la salud

Kingsley Nnanna Ukwaja, Isaac Alobu, Seye Abimbola, Philip Christy Hopewell

Resumen

Antecedentes: Los estudios sobre los costos en que incurren los pacientes para la atención de la tuberculosis (TB) están limitados ya que estos costos se reportan como promedios, y el efecto económico de los costos está estimado sobre la base de los ingresos promedio por paciente o por familia. Los gastos promedio no representan a los pobres debido a que ellos gastan menos en tratamientos en comparación con otros grupos socioeconómicos. Así, se desconoce la medida en que los gastos para la atención de la TB tienen el peligro de llevar a las familias a la pobreza (o de empobrecerlas aún más) y sus determinantes. Evaluamos la incidencia y las determinantes de los pagos catastróficos de las familias para la atención de la TB en las regiones rurales de Nigeria.

Métodos: Se obtuvieron datos de una encuesta de 452 pacientes de TB pulmonar muestreados de tres establecimientos médicos del Estado de Ebonyi en Nigeria. Empleando costos directos y datos de ingresos, analizamos la incidencia de pagos catastróficos de las familias utilizando como umbrales el tradicional >10% del ingreso familiar y el ≥40% de ingresos no para comida, como recomienda la Organización Mundial de la Salud. Empleamos el análisis logístico de regresión para identificar las determinantes de los pagos catastróficos.

Resultados: Los costos directos por familia para atención médica de la TB fueron US$157 o 14% del ingreso anual promedio. La incidencia de pagos catastróficos fue 44%; con 69% y 15% de los cuartiles de ingresos familiares más pobres y más ricos que experimentaron actividad catastrófica, respectivamente. Las determinantes independientes de pagos catastróficos fueron: edad >40 años (razón de probabilidad ajustada [aOR] 3.9; 95% intervalo de confianza [CI], 2.0, 7.8), sexo masculino (aOR 3.0; CI 1.8, 5.2), residencia urbana (aOR 3.8; CI 1.9, 7.7), educación formal (aOR 4.7; CI 2.5, 8.9), atención en un establecimiento particular (aOR 2.9; 1.5, 5.9), familia pobre (aOR 6.7; CI 3.7, 12), familia en la que el paciente es el sostén principal (aOR 3.8; CI 2.2, 6.6]), y coinfección de VIH (aOR 3.1; CI 1.7, 5.6).

Conclusión: Las estrategias actuales para reducir los costos no son suficientes para impedir que las familias incurran en pagos catastróficos de su propio bolsillo para la atención médica de la TB. Se necesitan intervenciones económicas y sociales para identificar los grupos en riesgo, y las intervenciones a nivel de la comunidad pueden reducir las ineficiencias en el camino de la búsqueda de atención médica. Estas observaciones deben proporcionar información para las estrategias para la TB después de 2015 e influir en la elaboración de las políticas de los servicios de salud que tienen el objeto de ser gratuitos.

Translated from English version into Spanish by Miguel Carmona, through

TRANSLATORS
WITHOUT BORDERS
